# Multidisciplinary treatment for patients with stage IV gastric cancer: the role of conversion surgery following chemotherapy

**DOI:** 10.1186/s12885-018-4998-x

**Published:** 2018-11-15

**Authors:** Seung-Hoon Beom, Yoon Young Choi, Song-Ee Baek, Shuang-Xi Li, Joon Seok Lim, Taeil Son, Hyoung-Il Kim, Jae-Ho Cheong, Woo Jin Hyung, Seung Ho Choi, Minkyu Jung, Hyo Song Kim, Hei-Cheul Jeung, Hyun Cheol Chung, Sun Young Rha, Sung Hoon Noh

**Affiliations:** 1 Department of Internal Medicine, Yonsei Cancer Center, Yonsei University College of Medicine, 50–1 Yonsei-ro, Seodaemun-gu, Seoul, 120–752 Republic of Korea; 2 Department of Surgery, Yonsei Cancer Center, Yonsei University College of Medicine, 50–1 Yonsei-ro, Seodaemun-gu, Seoul, 120–752 Republic of Korea; 3 Department of Radiology, Yonsei University College of Medicine, 50–1 Yonsei-ro, Seodaemun-gu, Seoul, 120–752 Republic of Korea; 4 Key laboratory of Carcinogenesis and Translational Research (Ministry of Education), Department of Gastrointestinal Surgery, Peking University Cancer Hospital & Institute, Beijing, 100142 China

**Keywords:** Gastric cancer, Metastasis, Gastrectomy, Chemotherapy, Conversion surgery

## Abstract

**Background:**

With advances in gastric cancer chemotherapy, conversion surgery has drawn attention as a new strategy to improve the outcome of stage IV disease. We investigated the efficacy of conversion surgery following chemotherapy for patients with stage IV gastric cancer.

**Methods:**

We retrospectively reviewed clinico-pathologic variables and oncologic outcomes for 101 patients with stage IV gastric cancer who were treated with systemic chemotherapy followed by gastrectomy with intension of curative resection from January 2005 to December 2012.

**Results:**

In terms of the best response from palliative chemotherapy, complete or partial response were observed in 65 patients (64.4%) in overall. Complete response of metastatic site were observed in 72 (71.3%) and 66 (65.3%) patients as best and pre-operative response, respectively. The overall complete macroscopic resection, rate was 56.4%. Eleven patients (10.9%) received combined metastasectomy. There was no postoperative surgery-related mortality for 1 month. The median overall survival time was 26.0 months. Multivariable analysis identified complete macroscopic resection, chemotherapy response (complete response/partial response) of metastatic sites, and change in CEA level as independent prognostic factors contributing to overall survival.

**Conclusions:**

Patients with stage IV gastric cancer who exhibit a good clinical response to chemotherapy might obtain greater survival benefit from gastrectomy following chemotherapy compared with patients who exhibit a poor response to chemotherapy. Prospective, randomized trials are required to determine the best strategy for combining initial chemotherapy with subsequent gastrectomy.

**Electronic supplementary material:**

The online version of this article (10.1186/s12885-018-4998-x) contains supplementary material, which is available to authorized users.

## Background

Despite a declining incidence in many developed countries, gastric cancer remains the second most common cause of cancer-related deaths in the world [1, 2]. Although the prognosis of metastatic gastric cancer is poor, combination chemotherapy improved the quality of life and overall survival (OS) compared with the best supportive care in several randomized studies [3, 4]. In general, 5-fluorouracil (5-FU)-based or cisplatin-based combination regimens are widely accepted as potential standard therapies with a response rate of around 30–40% and median OS of 9–11 months [5, 6]. More recently, even greater progress has been achieved by combining conventional chemotherapy with a targeted monoclonal antibody (median OS, 13.8 months) [7]. Although a large proportion of patients with metastatic or recurrent gastric cancer respond initially to chemotherapy, the disease ultimately progresses. In addition, a substantial proportion of patients have primary refractory diseases. Therefore, novel therapeutic strategies for treating metastatic gastric cancer are required.

Currently, surgery is not a standard treatment option for patients with gastric cancer with distant metastasis, except for those who need palliative surgery for bleeding, obstruction, or perforation caused by the tumor. Recently, a randomized, controlled trial of reduction surgery plus chemotherapy versus chemotherapy alone for M1 gastric cancer (REGATTA trial) failed to show any efficacy of surgery [8]. Recent advances in chemotherapy for gastric cancer have raised new clinical questions regarding the role of surgical intervention for patients with a good chemotherapy response, even if the patients have distant metastasis. Surgery for those patients might provide long-term survival benefit by removing macroscopic lesions remaining after chemotherapy. That type of surgery, referred to as conversion surgery, aims to cure the disease, rather than just provide palliative treatment, on the basis of the response to chemotherapy, as is done for initially unresectable colorectal cancer [9]. However, the clinical value of such multimodal therapy combining chemotherapy and conversion surgery for stage IV gastric cancer remains controversial.

The aim of this study was to evaluate the efficacy and feasibility of surgery in patients with stage IV gastric cancer who were treated by gastrectomy following chemotherapy, with a particular focus on the selection of patients who might benefit from conversion surgery.

## Methods

### Patients

We retrospectively identified patients with a clinical diagnosis of stage IV gastric cancer who underwent gastrectomy following chemotherapy in Yonsei Cancer Center from January 2005 through December 2012. The key eligibility criteria were: (i) patients with histologically proven gastric cancer, (ii) who received systemic chemotherapy at the time of diagnosis of stage IV disease according to the AJCC 7th staging system, and (iii) underwent subsequent gastrectomy with the intention of curative resection. Consecutive patients corresponding to those criteria were included. We initially identified 106 patients who underwent gastrectomy following chemotherapy. Four patients were excluded because the aim of surgery was palliative (two patients underwent gastrectomy because of bleeding, and two patients did so because of gastric obstruction). One additional patient who received initial chemotherapy at another institution was excluded from the analysis, because there were no available clinical data from the time of diagnosis. Thus, we analyzed 101 patients (Additional file 1: Figure S1). This study was approved by the Institutional Review Board of Severance Hospital, Yonsei University College of Medicine (4–2016-0408).

### Variables for analyses

We grouped the variables collected for study into four categories: baseline characteristics, chemotherapy-related information, variables before operation, and surgical outcomes including pathologic results. The baseline characteristics included age, gender, clinical T and N stage, type of distant metastasis, neutrophil / lymphocyte ratio (NLR, %), systemic immune-inflammation index (SII, platelet count × neutrophil count / lymphocyte count), and albumin level (g/dL) at initial diagnosis. The chemotherapy-related information included the regimen of palliative chemotherapy; the best response and preoperative response (response at the time before operation) to palliative chemotherapy overall, at the local region, and at the metastatic sites; number of cycles of chemotherapy. The response to chemotherapy was defined according to RECIST criteria version 1.1 and categorized as complete response (CR), partial response (PR), stable disease (SD), or progressive disease (PD) [10]. The variables before operation included the change in body mass index (BMI), the change in tumor markers, the clinical T and N stage immediately prior to surgery, and during the time interval between the initial diagnosis and surgery. Clinical T and N stage was evaluated by one radiologist with CT images according to AJCC 7th guideline. We assumed stomach lesion with definite extramural fat infiltration or adjacent organ invasion as clinical T4 lesion. The patients were classified into three categories based on BMI (kg/m^2^) in the period between diagnosis and surgery: underweight (BMI < 17.5), overweight (BMI ≥ 23.0), and normal (17.5 ≤ BMI < 23.0). The changes in the carcinoembryonic antigen (CEA) and carbohydrate antigen (CA 19–9) tumor markers between the initial diagnosis and surgery were classified as stable (no change), improved (high levels decreased to the normal range), worse (normal levels increased to the high range), and not available (with any missing value). The surgical outcomes included the presence of peritoneal infiltration, whether or not metastasectomy was performed, the extent of gastrectomy and lymph node (LN) dissection, and curative resection. In this study, curative resection means complete macroscopic resection (CMR) which was defined there was no visible tumor in abdomen after surgery. We also considered variables of the pathologic T and N stage, the Lauren classification of the surgical specimen, and presence of postoperative chemotherapy as potential prognostic factors.

### Statistical analysis

We analyzed categorical variables based on the proportions among the patients and continuous variables based on the mean and standard deviation. The primary outcome was OS, defined as the time from the diagnosis of gastric cancer to the time of death by any cause. We generated survival curves by the Kaplan-Meier method and compared them by log-rank tests. We used a Cox proportional hazard regression model to identify risk factors for OS. We described the risk factors by a hazard ratio (HR) with 95% confidence interval (CI). We conducted a multivariable Cox analysis with the variables that had *p*-value ≤0.10 in the univariable analyses. We selected the final model by the forward likelihood ratio method. Two-tailed *p*-value ≤0.05 was considered statistically significant. All analyses were conducted using SPSS (version 19.0 software, IBM SPSS, Chicago, IL).

## Results

### Characteristics of the patients

The median age of the enrolled patients was 52 years (Table 1). The study population was 59.4% male. At the time of diagnosis, 80.2% of the patients were suspected to have clinically serosa-positive depth of invasion, and 84.2% of the patients were suspected to be beyond the clinical N1 stage. The reasons for diagnosis of stage IV disease varied, including peritoneal carcinomatosis (32.7%), liver metastasis (10.9%), distant LN metastasis (34.7%), Krukenberg tumor (20.0%), and two or more distant metastases (19.8%). The mean baseline values of NLR, SII, and albumin were 2.67, 9.14, and 3.94, respectively.

**Table 1:** Characteristics of the enrolled patients (*n* = 101)

Baseline Characteristics		Chemotherapy		Preoperative		Surgical Outcomes	
Age, median, years	52 (range 26–78)	Regimen		BMI		Peritoneal infiltration	
Gender		Platinum + FU	51 (50.5)	Normal	39 (38.6)	No	69 (68.3)
Male	60 (59.4)	Taxane + FU	12 (11.9)	Under weight	9 (8.9)	Yes	32 (31.7)
Female	41 (40.6)	Platinum + Taxane + FU	15 (14.9)	Over weight	53 (52.5)	Metastasectomy	
cT stage		Taxane + Platinum	16 (15.8)	Change of CEA level		No	90 (89.1)
Serosa negative	20 (19.8)	Others	7 (6.9)	Stable	66 (65.3)	Yes	11 (10.9)
Serosa positive	81 (80.2)	Best response		Improved	16 (15.8)	Hepatectomy	3
cN stage		Overall		Worse	3 (3.0)	#16	6
N0/N1	16 (15.8)	CR/PR	65 (64.4)	NA	16 (15.8)	Oophorectomy	3
N2/N3	85 (84.2)	SD/PD	36 (35.6)	Change of CA19–9 level		Extent of gastrectomy	
Type of distant metastasis		Local region		Stable	66 (65.3)	TG	57 (56.4)
Peritoneal carcinomatosis	33 (32.7)	CR/PR	47 (46.5)	Improved	10 (9.9)	DG	44 (43.6)
Liver metastasis	11 (10.9)	SD/PD	54 (53.5)	Worse	5 (5.0)	Extent of LND	
Distant LN metastasis	35 (34.7)	Metastatic site		NA	20 (19.8)	< D2	25 (24.8)
Krukenberg tumor	2 (2.0)	CR/PR	84 (83.2)	Pre_op cT stage		≥ D2	76 (75.2)
Any combination	20 (19.8)	SD/PD	17 (16.8)	Serosa negative	48 (47.5)	Complete macroscopic resection	
NLR (Neut/lympho)	2.67 ± 1.30	Preoperative response		Serosa positive	53 (52.5)	Yes	57 (56.4)
SII (PLT*Neut/lympho)	9.14 ± 6.88	Overall		Pre_op cN stage		No	44 (43.6)
Albumin (g/dL)	3.94 ± 0.53	CR/PR	60 (59.4)	N0/N1	33 (32.7)	pT stage	
		SD/PD	41 (40.6)	N2/N3	68 (67.3)	Serosa negative (pT1–3)	47 (46.5)
		Local region		Interval to surgery		Serosa positive (pT4a/b)	54 (53.5)
		CR/PR	43 (42.6)	≤ 24 weeks	51 (50.5)	pN stage	
		SD/PD	58 (57.4)	> 24 weeks	50 (49.5)	pN0/N1	35 (34.7)
		Metastatic site				pN2/N3	66 (65.3)
		CR/PR	78 (77.2)			Lauren classification	
		SD/PD	23 (22.8)			Intestinal	46 (45.5)
		Number of Cycles of Chemotherapy	6 (range 1–32)			Diffuse	42 (41.6)
		≤ 6 cycles	62 (61.4)			Others	13 (12.9)
		> 6 cycles	39 (38.6)			Postoperative chemotherapy	
						No	17 (16.8)
						Yes	84 (83.2)

*LN* lymph node, *NLR* neutrophil lymphocyte ratio, *SII* systematic immune-inflammation index, *CR* complete response, *PR* partial response, *SD* stable disease, *PD* progressive disease, *BMI* body mass index, *CEA* carcinoembryonic antigen, *CA19–9* carbohydrate antigen 19–9, *TG* total gastrectomy, *DG* distal gastrectomy, *LND* lymph node dissection, *FU* fluoropyrimidine

The preoperative chemotherapy regimen was platinum plus fluoropyrimidine in 50.5% of the patients, taxane plus fluoropyrimidine in 11.9% of the patients, platinum plus taxane plus fluoropyrimidine in 14.9% of the patients, taxane plus platinum in 15.8% of the patients, and others in 6.9% of the patients. Overall, 64.4 and 59.4% of the patients had CR or PR to preoperative chemotherapy as best response and preoperative response to the chemotherapy, respectively. When the responses were divided into the local region and the metastatic sites, the rate of CR/PR was lower in the local region (46.5 and 42.6%) than in the metastatic sites (83.2 and 77.2%) in both best response and preoperative response. CR of metastatic site was observed in 72 (71.3%) and 66 (65.3%) patients in best and preoperative response, respectively. The median number of cycles of chemotherapy was 6 (1–32 cycles).

Nine patients (8.9%) were considered underweight during chemotherapy treatment, and 52.5% of the patients were considered overweight. The levels of CEA and CA19–9 improved in 16 and 10 patients, respectively, and worsened in 3 and 5 patients, respectively. After the initial chemotherapy, both the T and the N staging was downgraded in overall; the proportion of serosa-negative was 47.5% and that of N0/N1 was 32.7%. The interval from chemotherapy to surgery was ≤24 weeks in 51 patients.

During the surgery, 32 patients displayed peritoneal infiltration. Metastasectomy was conducted for 11 patients (three hepatectomy, six para-aortic LN dissection, and three oophorectomy). Fifty-seven patients underwent total gastrectomy, and 76 patients underwent ≥ D2 LN dissection. Finally, CMR was achieved in 57 patients (56.4%), and 46 and 42 patients were found to have intestinal and diffuse-type gastric cancer, respectively. In the final pathologic diagnosis, 54 (53.5%) of the patients were serosa positive (pT4a/b), and 66 of the patients (65.3%) had ≥3 metastatic LNs. Eighty-four patients were treated with postoperative chemotherapy. There was no postoperative surgery-related mortality for 1 month. The detailed information is summarized in Table 1.

### Prognostic factors in the population

**Fig. 1:**
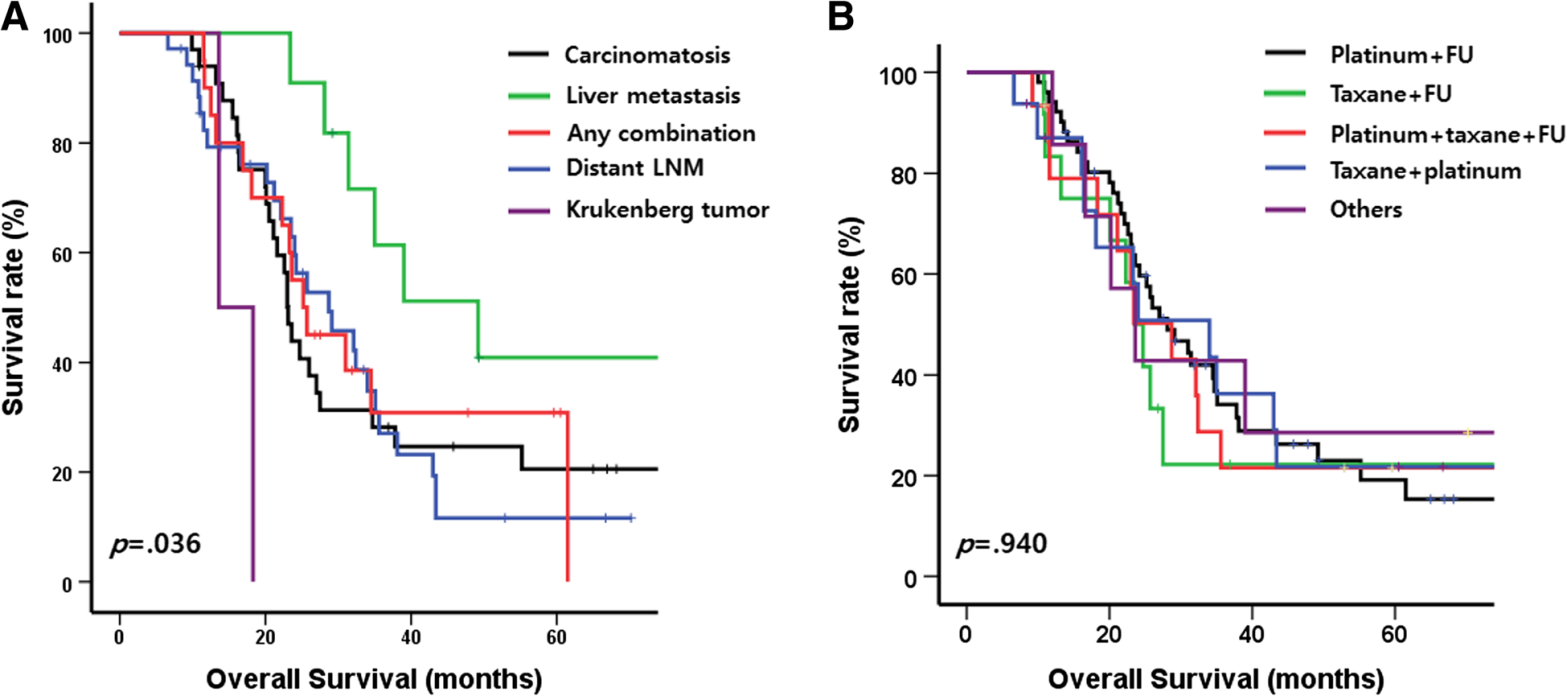
Kaplan-Meier curves with log-rank test for overall survival of the patients with stage IV gastric cancer who treated by gastrectomy following chemotherapy **a**) according to the type of distant metastasis, **b**) and chemotherapy regimen

The median follow-up duration was 63.3 months. Seventy-three patients (72.3%) died during follow up. The median OS of the patients was 26.0 (95% CI, 21.6–30.3) months. Figure 1a depicts the different prognoses by the type of distant metastasis. Compared with the prognoses for patients with other types of distant metastasis, the prognosis for patients with liver metastasis (median survival, 49.2 months) was better, and that for patients with Krukenberg tumor (median survival, 13.6 months) was worse. In the univariate Cox analyses, greater age was related to better OS (*p* = 0.047), whereas other baseline characteristics such as gender, initial clinical T and N stage, and initial NLR, SII, and albumin levels were not statistically significant (Table 2).

**Table 2:** Univariate Cox proportional hazard model of overall survival by each variable

Baseline Characteristics	HR (95% CI)	*p*-value	Chemotherapy	HR (95% CI)	*p*-value
Age	0.978 (0.958–1.000)	**0.047**	Regimen		0.941
Gender		0.111	Platinum + FU	1	
Male	1		Taxane + FU	1.336 (0.643–2.776)	0.437
Female	1.464 (0.916–2.339)		Platinum + Taxane + FU	1.116 (0.569–2.188)	0.750
cT stage		0.734	Taxane + Platinum	1.003 (0.511–1.969)	0.994
Serosa negative	1		Others	0.904 (0.354–2.306)	0.832
Serosa positive	1.107 (0.616–1.988)		Best response		
cN stage		0.743	Overall		0.201
N0/N1	1		CR/PR	1	
N2/N3	1.109 (0.597–2.061)		SD/PD	1.359 (0.849–2.175)	
Type of distant metastasis		0.065	Local region		0.576
Peritoneal carcinomatosis	1		CR/PR	1	
Liver metastasis	0.407 (0.166–0.994)	0.049	SD/PD	1.142 (0.717–1.817)	
Distant LN metastasis	1.025 (0.590–1.780)	0.930	Metastatic site		**0.013**
Krukenberg tumor	4.423 (1.004–19.487)	0.049	CR/PR	1	
Any combination	0.900 (0.467–1.735)	0.753	SD/PD	2.105 (1.171–3.783)	
NLR (Neut/lympho)	1.349 (0.837–2.177)	0.219	Preop. response		
SII (PLT*Neut/lympho)	1.252 (0.776–2.019)	0.357	Overall		0.635
Albumin		0.986	CR/PR	1	
low	1		SD/PD	1.119 (0.704–1.777)	
normal	1.006 (0.480–2.108)		Local region		0.875
			CR/PR	1	
			SD/PD	0.963 (0.602–1.540)	
			Metastatic site		**0.020**
			CR/PR	1	
			SD/PD	1.850 (1.103–3.103)	
			Number of Cycles of Chemotherapy	0.970 (0.930–1.013)	0.167
			≤ 6 cycles	1	0.746
			> 6 cycles	0.925 (0.578–1.480)	
Pre-Operation	HR (95% CI)	*p-*value	Surgical Outcomes	HR (95% CI)	*p*-value
BMI		**0.004**	Peritoneal infiltration		**0.002**
Normal	1		No	1	
Underweight	3.538 (1.607–7.789)	**0.002**	Yes	2.181 (1.343–3.540)	
Overweight & obese	1.073 (0.647–1.778)	0.786	Metastasectomy		0.252
Change in CEA level		**0.009**	No	1	
Stable	1		Yes	1.507 (0.747–3.039)	
Improved	0.410 (0.193–0.868)	**0.020**	Extent of gastrectomy		**0.008**
Worse	4.174 (1.255–13.881)	**0.020**	TG	1	
NA	0.856 (0.446–1.646)	0.642	DG	0.521 (0.322–0.842)	
Change in CA19–9 level		0.399	Extent of LND		0.402
Stable	1		< D2	1	
Improved	1.020 (0.437–2.381)	0.963	≥ D2	0.802 (0.480–1.343)	
Worse	0.907 (0.282–2.911)	0.869	Complete Macroscopic Resection		**< 0.001**
NA	0.580 (0.309–1.089)	0.090	Yes	1	
Pre_op cT stage		0.069	No	2.348 (1.471–3.748)	
Serosa negative	1		pT stage		**0.002**
Serosa positive	1.537 (0.967–2.444)		Serosa negative (pT1–3)	1	
Pre_op cN stage		0.567	Serosa positive (pT4a/b)	2.109 (1.311–3.393)	
N0/N1	1		pN stage		0.121
N2/N3	1.155 (0.705–1.894)		pN0/N1	1	
Time interval to surgery		0.926	pN2/N3	1.480 (0.901–2.430)	
≤ 24 weeks	1		Lauren classification		**0.013**
> 24 weeks	0.926 (0.584–1.468)		Intestinal	1	
			Diffuse	1.960 (1.204–3.190)	**0.007**
			Others	0.867 (0.361–2.079)	0.748
			Postoperative chemotherapy		0.172
			No	1	
			Yes	1.629 (0.809–3.281)	

*LN* lymph node, *NLR* neutrophil lymphocyte ratio, *SII* systematic immune-inflammation index, *CR* complete response, *PR* partial response, *SD* stable disease, *PD* progressive disease, *BMI* body mass index, *CEA* carcinoembryonic antigen, *CA19–9* carbohydrate antigen 19–9, *TG* total gastrectomy, *DG* distal gastrectomy, *LND* lymph node dissection, *FU* fluoropyrimidine

The bold represents statistical significance

**Fig. 2:**
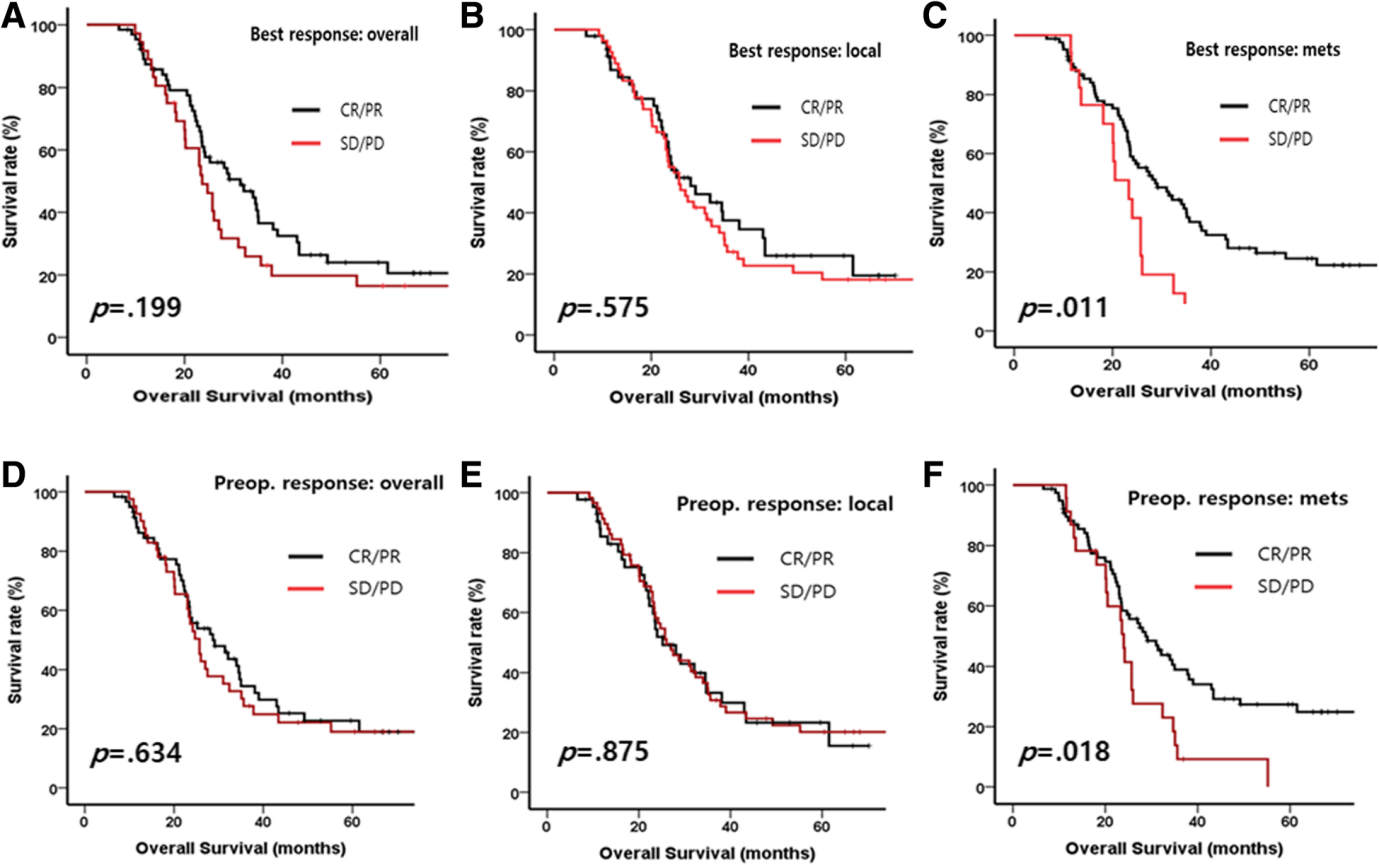
Kaplan-Meier curves with log-rank test for overall survival of the patients with stage IV gastric cancer who treated by gastrectomy following chemotherapy **a**) by overall response in best response of chemotherapy, **b**) by chemotherapy response of local region, **c**) by chemotherapy response of metastatic site, **d**) by overall response before operation, **e**) by response of local region before operation, **f**) by response of metastatic site before operation

There was no difference in prognosis based on the regimen of preoperative chemotherapy (Fig. 1b and Table 2). The overall response to chemotherapy was not statistically significant to the prognosis in both best and preoperative response (log-rank *p* = 0.199, 0.634, respectively, Fig. 2a and d). The response of local region to chemotherapy showed similar prognosis in both best and preoperative response (log-rank *p* = 0.575, 0.875, respectively, Fig. 2b and e). However, CR/PR at the metastatic sites was significantly related to OS in both best and preoperative response (log-rank *p* = 0.011 and 0.018, respectively, Fig. 2c and f) and univariate HRs were also statistically significant (HR for best response: 2.105 [1.171–3.783], *p* = 0.013, HR for preoperative response: 1.850 [1.103–3.103], *p* = 0.020, Table 2).

**Fig. 3:**
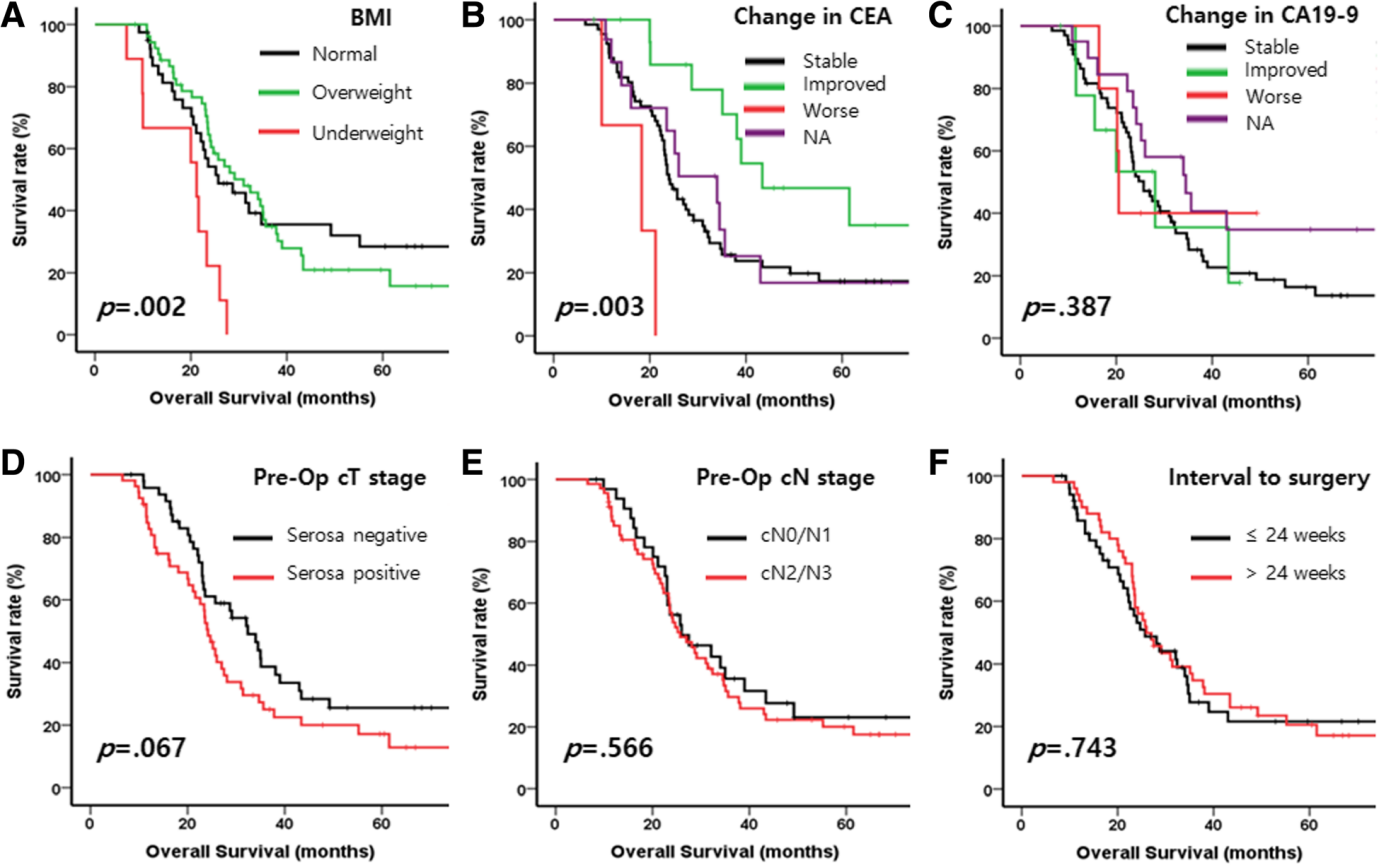
Kaplan-Meier curves with log-rank test for overall survival of the patients with stage IV gastric cancer who treated by gastrectomy following chemotherapy **a**) by BMI, **b**) by change of CEA level, **c**) by change of CA19–9 level, **d**) pre-operative clinical T stage, **e**) by pre-operative N stage, **f**) by time interval between diagnosis and surgery

Among the preoperative variables, BMI and changed CEA level was related to prognosis (Fig. 3a and b, and Table 2): patients experienced underweight were related to poor prognosis (*p* = 0.002) and patients with improved CEA level were related good prognosis (*p* = 0.003). Other variables such as changed CA19–9 level, preoperative clinical T and N stage, and interval to surgery were not related to the prognosis (Fig. 3c-f and Table 2).

**Fig. 4:**
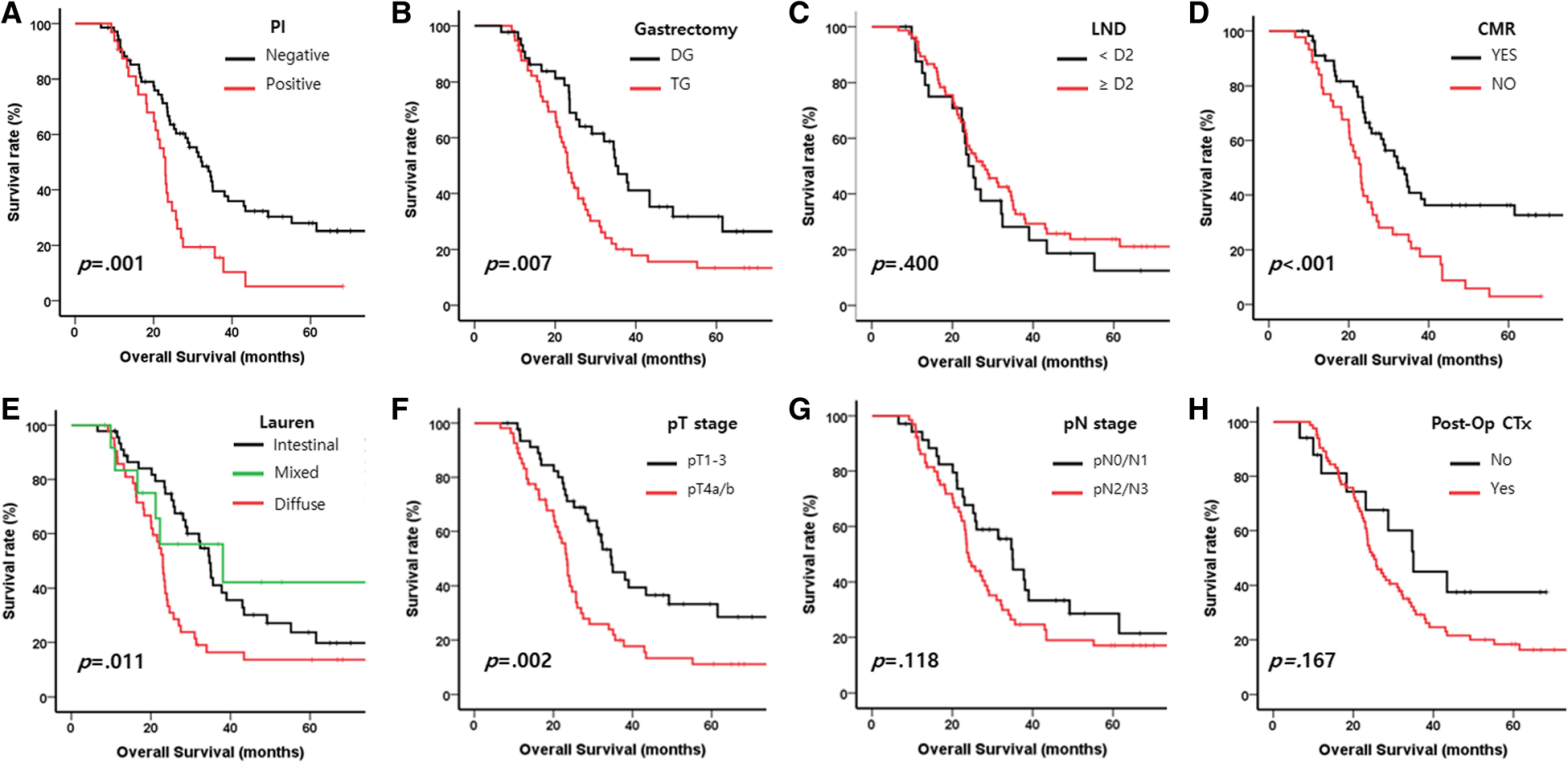
Kaplan-Meier curves with log-rank test for overall survival of the patients with stage IV gastric cancer who treated by gastrectomy following chemotherapy **a**) by the presence of peritoneal infiltration, **b**) by extent of gastrecotmy, **c**) by extent of lymph node dissection, **d**) by complete macroscopic resection or not, **e**) by Lauren classification, **f**) by pathologic T stage, **g**) by pathologic N stage, **h**) by post-operative chemotherapy or not

Figure 4 and Table 2 showed the prognosis of each variable of surgical outcomes. The presence of peritoneal infiltration (*p* = 0.001, Fig. 4a), extent of gastrectomy (*p* = 0.007, Fig. 4b), CMR (*p* < 0.001, Fig. 4d), pathologic T stage (*p* = 0.002, Fig. 4f), Lauren classification (*p* = 0.001, Fig. 4e) were related to the prognosis, and univariable Cox analysis showed similar results (Table 2). Other factors such as metastasectomy, extent of LN dissection, pathologic N stage, and postoperative chemotherapy were not related to OS (Fig. 4c, g, h and Table 2).

The multivariable analysis was conducted with the variables that had *p*-value < 0.1 in the univariate analyses (age, type of distant metastasis, best response of the metastatic sites, BMI, change in CEA level, preoperative cT stage, presence of peritoneal infiltration, extent of gastrectomy, CMR, pT stage, and Lauren classification). The best response of the metastatic sites [HR: 1.822 (0.999–3.323), *p* = 0.050], change in CEA level [HR of improved, worse, and NA compared with stable: 0.564 (0.259–1.226), *p* = 0.148; 5.013 (1.477–17.017), *p* = 0.010; 0.973 (0.504–1.877), *p* = 0.935; respectively; overall *p* = 0.024], and CMR [HR: 1.998 (1.233–3.238), *p* = 0.005] were selected as factors that related to the prognosis of the patients (Table 3).

**Table 3:** Multivariable Cox proportional hazard model of overall survival

Variables^a^	HR (95% CI)	*p*-value
Best response of metastatic site		**0.050**
CR/PR	1	
SD/PD	1.822 (0.999–3.323)	
Change of CEA level		**0.024**
Stable	1	
Improved	0.564 (0.259–1.226)	0.148
Worse	5.013 (1.477–17.017)	**0.010**
NA	0.973 (0.504–1.877)	0.935
Complete Macroscopic Resection		**0.005**
Yes	1	
No	1.998 (1.233–3.238)	

*CR* complete response, *PR* partial response, *SD* stable disease, *PD* progressive disease, *CEA* carcinoembryonic antigen

^a^the final model was selected by a forward likelihood ratio method from the variables with *p*-value < 0.10 in a univariate Cox proportional hazard model

The bold represents statistical significance

### Subgroup analyses by the type of distant metastasis

Subgroup analyses according to the type of distant metastasis were conducted to evaluate the effects of the variables that were selected for the final model (best response of metastatic sites, change in CEA level, and CMR). In the patients with peritoneal carcinomatosis, there was no trend for the best response of metastatic sites or change in CEA level (Additional file 2: Figure S2A and B), and CMR was marginally related to better survival but was not statistically significant (log-rank *p* = 0.064, Additional file 2: Figure S2C). There were no remarkable trends among the patients with liver metastasis (Additional file 3: Figure S3A-C) and distant LN metastasis (Additional file 4: Figure S4A-C), respectively. Among the patients with two or more distant metastases, the best response of the metastatic sites was related to OS (log-rank *p* = 0.007, Additional file 5: Figure S5A), and there were trends for better prognosis with improved CEA level and CMR, respectively, although those trends were not statistically significant (Additional file 5: Figure S5B and C).

## Discussion

Patients with stage IV gastric cancer usually have a poor prognosis and are primarily considered for systemic chemotherapy, but not for surgery. The exception to that is patients who require rapid palliation of cancer-related complications such as bleeding or obstruction [11]. Recent advances in gastric-cancer chemotherapy, including the introduction of new anticancer agents and the development of multi-agent regimens, have made complete macroscopic resection possible in some patients with stage IV gastric cancer. That strategy of treatment is referred to as conversion gastrectomy with curative intent and is distinct from palliative gastrectomy. The results of previous studies of surgical resection after preoperative chemotherapy for initially metastatic gastric cancer are summarized in Table 4 [12–24]. Although the chemotherapy regimens and definitions of gastrectomy varied across the studies, the reported OS ranged 19 to 53 months, which revealed much better outcomes, considering the generally poor prognosis for stage IV gastric cancer. The limitations of the previous studies include small study samples and the use of retrospective methods of analysis. Some of the studies investigated prognostic factors for gastrectomy and presented various factors including R0 resection as significant prognostic factors.

**Table 4:** Summary the results of gastrectomy following systemic chemotherapy for stage IV gastric cancer

Reference	Author	Year	Metastasis	Regimen of chemotherapy	Patients who underwent gastrectomy		Whole study population (if it included non-surgery cases)		Prognostic factors
					Number of patients	MST (months)	Number of patients	MST (months)	
12	Nakajima et al.	1997	M1	FLEP (5-Fluorouracil, Leucovorin, Cisplatin, Etoposide)	19	NA	30	6.5	NA
13	Yano et al.	2002	unresectable (M0 + M1)	FEMTXP (5-Fluorouracil, Epirubicin, Methotrexate, Cisplatin) or THP-FLPM (Pirarubicin,5-Fluorouracil, Leucovorin, Cisplatin, Mitomycin C)	14	NA	33	NA	salvage surgery
14	Satoh et al.	2006	M0 + M1	S-1, Cisplatin	36	NA	45	21.8	NA
15	Ishigami et al.	2008	M1	Paclitaxel, S-1	18	25.7	–	–	R0 resection
16	Okabe et al.	2009	M1 (peritoneal metastasis)	S-1, Cisplatin	32	NA	41	20.4	NA
17	Suzuki et al.	2010	unresectable (M0 + M1)	Docetaxel, S-1	20	28.5	–	–	NA
18	Kanda et al.	2012	M1	S-1 based chemotherapy	28	29.0	–	–	histological tumor length (< 5 cm vs. ≥ 5 cm)
19	Satoh et al.	2012	M1	S-1, Cisplatin	44	NA	51	19.2	NA
20	Han et al.	2013	M1	Various	34	22.9 (R0 resection) and 7.8 (non-R0 resection)	–	–	ypN stage (N0–2 vs. N3)
21	Yabusaki et al.	2013	M1	S-1, Cisplatin	97	22.5	148	16.8	surgery, R0 resection, D2/D3 lymph node dissection, CR/PR response
22	Fukuchi et al.	2015	unresectable (M0 + M1)	S-1, Cisplatin or S-1, Paclitaxel	40	53.0	151	16.0	one non-curative factor, R0 resection
23	Kinoshita et al.	2015	M1	Docetaxel, Cisplatin, S-1	34	29.9	57	20.9	potential resectability
24	Ito et al.	2015	M1	Various	14	24.8	70	14.1	NA
Present study			M1	Various	101	26.0	–	–	curative resection (complete macroscopic resection), chemotherapy response (CR/PR) of metastatic site, change of CEA level

*MST* median survival time, *CR* complete response, *PR* partial response, *SD* stable disease, *PD* progressive disease, *NA* not available, *CEA* carcinoembryonic antigen

In our study, the median OS of the patients was 26.0 months. Survival outcomes did not differ significantly according to clinical factors at diagnosis including metastatic sites and chemotherapy regimens. In addition to CMR, CEA change as well as the response of metastatic sites to chemotherapy were significant prognostic factors in the multivariable analysis.

Successful curative surgical resection is the most frequently presented prognostic factor of conversion gastrectomy so far. Yabusaki et al. reported that patients obtaining CR/PR to chemotherapy as well as those with R0 resection and D2/D3 LN dissection had longer survival [21]. Fukuchi et al. also demonstrated that R0 resection as well as one non-curative factor was an independent significant predictor for overall survival in patients who underwent conversion surgery [22]. In general, R0 resection which means that no cancerous cell is seen microscopically in surgical specimen corresponds to resection for cure or complete remission. However, definitive R0 resection is rarely achieved because many cases with stage IV gastric cancer have non-measurable lesions including peritoneal diseases. For example, peritonectomy or pathologic confirmation of residual cancer in surgery for this case is not common surgical procedure in operative field. Our data showed that only 11(10.9%) of 101 patients underwent metastasectomy with gastrectomy. Thus we defined that curative resection means complete macroscopic resection in abdomen after surgery. Definition of the concept of “conversion therapy” and the patients who are eligible for such a procedure remain to be clarified.

In this study, the response to chemotherapy was important to the prognosis following surgery. Our data showed that the response of the metastatic sites, rather than the overall response, to chemotherapy was a significant prognostic factor. The reason could be related to the original nature of the RECIST evaluation system. We analyzed the chemotherapy response by dividing the response into that of the local region and that of the metastatic sites, adding those responses to the overall tumor response. The local tumor, including the primary stomach cancer and the regional LNs, is mostly evaluated as a non-measurable lesion, and its response is categorized as CR, non-CR/non-PD, or PD. CR of the primary stomach cancer is seldom observed. For example, although metastatic sites without measurable lesion reveal CR, the overall response is not CR or PR but is instead non-CR/non-PD in cases in which the local tumor without measurable lesion showed a non-CR/non-PD response. Our results suggest that the chemotherapy response of the metastatic sites is more important than the overall response for the selection of patients who would benefit from conversion gastrectomy.

Our results demonstrate that, in addition to the tumor response assessed grossly, the biochemical tumor response is another significant prognostic factor for OS. Patients with an improved CEA level might obtain a survival benefit from curative surgery subsequent to chemotherapy. In terms of the optimal timing of the surgery, we investigated whether the time interval between chemotherapy and surgery could influence the overall outcome, but the timing was not significant in a univariate analysis.

Recently, the REGATTA trial demonstrated that chemotherapy following palliative surgery for stage IV gastric cancer is not beneficial to survival outcomes based on an interim analysis and the trial was early closed [8]. Surgical resection following chemotherapy has since drawn attention from clinicians who treat patients with gastric cancer to enhance survival. It remains unclear whether conversion gastrectomy improves the prognosis in initially stage IV gastric cancer. The results of our large-scale study combined with those of previous studies demonstrate that conversion surgery after preoperative chemotherapy greatly extended survival for patients with stage IV gastric cancer. Patients who received CMR, showed CR/PR of metastatic sites to chemotherapy, and had an improved CEA level during preoperative treatment could have considerably better prognosis than those without those factors, suggesting that those factors might be useful selection criteria for conversion surgery. Further investigation is required, however, to determine how to maximize the chemotherapy response.

Our study has some limitations. First, we used a retrospective design to study patients at a single institute. Because of the retrospective design, the preoperative chemotherapy regimen and the timing of the operation varied somewhat. Second, the patients included in our study did not represent the whole population of patients with stage IV gastric cancer. Many of the patients who received surgery were likely to have potentially resectable disease at the time of diagnosis, and there might have been a selection bias for patients who respond well to chemotherapy. In addition, only small percent of patients could be received surgery among overall patients with stage IV gastric cancer in that period, therefore, the interpretation of benefit from conversion surgery in the present result should be cautious. Third, there might be a discrepancy between the clinical stage and the true disease dissemination, because metastasis was not pathologically confirmed in most of the patients. Lastly, type II error have to be considered for some subgroup analysis because of small size of cohort in this study.

We evaluated the outcomes of surgery for stage IV gastric cancer in the largest patient group to date and demonstrated that treatment with chemotherapy followed by gastrectomy is feasible and improved long-term survival. Better response (CR/PR) of metastatic sites to chemotherapy, change of CEA level, and CMR were significant prognostic factors.

## Conclusion

In conclusion, although stage IV gastric cancer still has a poor prognosis, our results suggest a practical, multidisciplinary treatment plan for select patients. Large-scale, prospective, multicenter, randomized trials are needed to further determine the best treatment strategy and to elucidate the prognostic role of conversion gastrectomy. Numerous obstacles are yet to be resolved regarding the selection of appropriate patients for conversion gastrectomy, the choice of preoperative/postoperative chemotherapy regimen for obtaining a maximal response, and the optimal timing of conversion surgery.

## Additional files

Additional file 1: **Figure S1.** Flow chart of patients’ recruitment. (TIF 75 kb)

Additional file 2: **Figure S2.** Kaplan-Meier curves with log-rank test for overall survival of the patients with peritoneal carcinomatosis by A) by chemotherapy response of metastatic site of, B) change of CEA level, C) complete macroscopic resection or not. (TIF 257 kb)

Additional file 3: **Figure S3.** Kaplan-Meier curves with log-rank test for overall survival of the patients with liver metastasis by A) by chemotherapy response of metastatic site, B) change of CEA level, C) complete macroscopic resection or not. (TIF 235 kb)

Additional file 4: **Figure S4.** Kaplan-Meier curves with log-rank test for overall survival of the patients with distant lymph node metastasis by A) by chemotherapy response of metastatic, B) change of CEA level, C) complete macroscopic resection or not. (TIF 291 kb)

Additional file 5: **Figure S5.** Kaplan-Meier curves with log-rank test for overall survival of the patients with two or more distant metastasis by A) by chemotherapy response of metastatic site, B) change of CEA level, C) complete macroscopic resection or not. (TIF 452 kb)

## Abbreviations

5-FU: 5-fluorouracil
BMI: Body mass index
CA 19–9: Carbohydrate antigen
CEA: Carcinoembryonic antigen
CI: Confidence interval
CMR: Complete macroscopic resection
CR: Complete response
HR: Hazard ratio
LN: Lymph node
NLR: Neutrophil lymphocyte ratio
OS: Overall survival
PD: Progressive disease
PR: Partial response
SD: Stable disease
SII: Systemic immune-inflammation index
