# Supplementary figures and images for: Multidisciplinary treatment for patients with stage IV gastric cancer: the role of conversion surgery following chemotherapy
Source: BMC Cancer. 2018 Nov 15;18:1116. doi: 10.1186/s12885-018-4998-x (PMC6238319; doi:10.1186/s12885-018-4998-x)

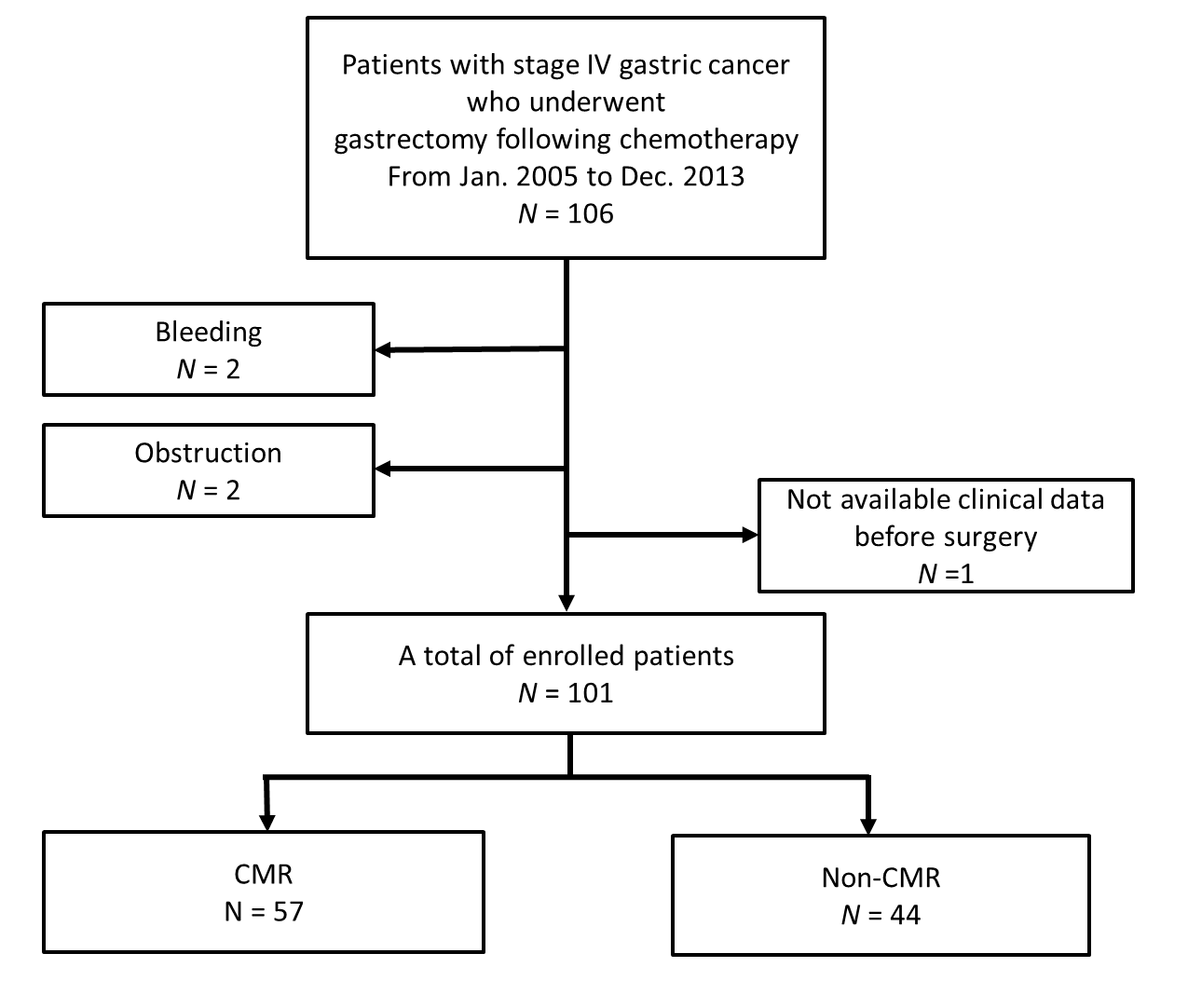

Supplement: Supplementary file 1 — Figure S1. Flow chart of patients’ recruitment. (TIF 75 kb) [file 12885_2018_4998_MOESM1_ESM.tif]

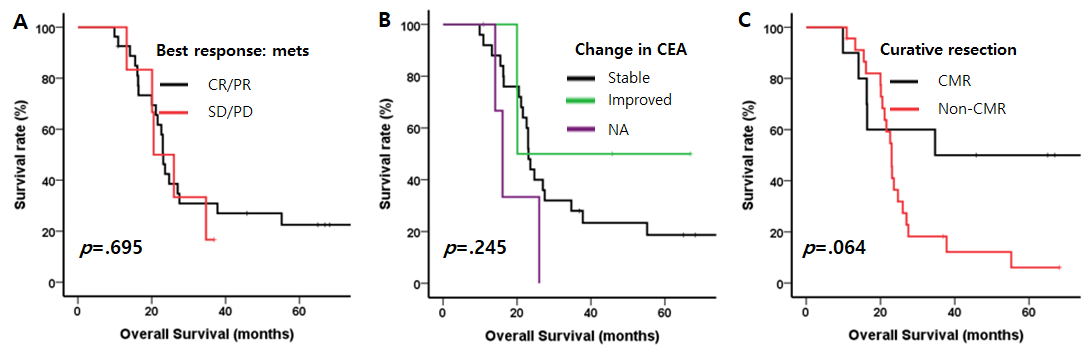

Supplement: Supplementary file 2 — Figure S2. Kaplan-Meier curves with log-rank test for overall survival of the patients with peritoneal carcinomatosis by A) by chemotherapy response of metastatic site of, B) change of CEA level, C) complete macroscopic resection or not. (TIF 257 kb) [file 12885_2018_4998_MOESM2_ESM.tif]

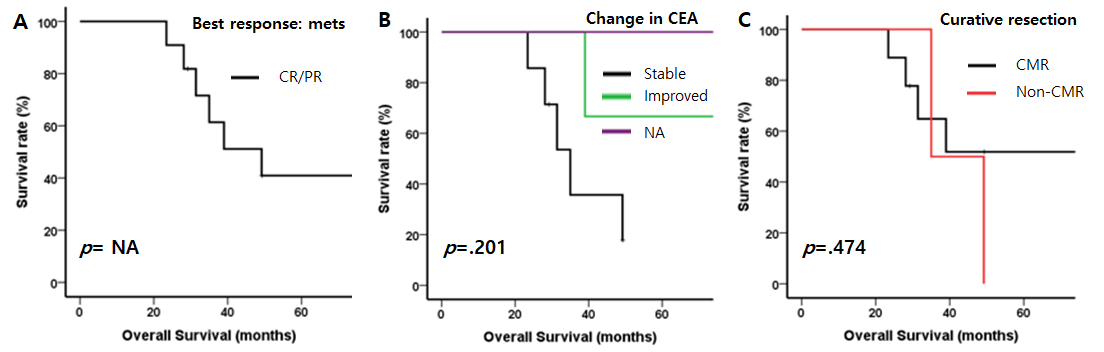

Supplement: Supplementary file 3 — Figure S3. Kaplan-Meier curves with log-rank test for overall survival of the patients with liver metastasis by A) by chemotherapy response of metastatic site, B) change of CEA level, C) complete macroscopic resection or not. (TIF 235 kb) [file 12885_2018_4998_MOESM3_ESM.tif]

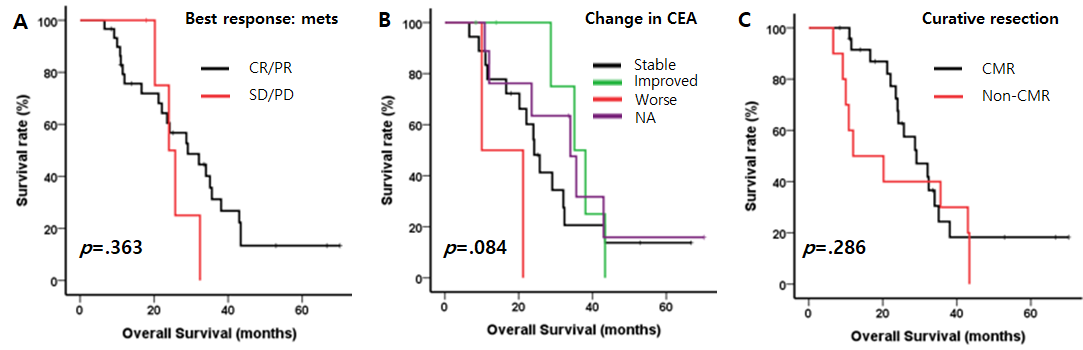

Supplement: Supplementary file 4 — Figure S4. Kaplan-Meier curves with log-rank test for overall survival of the patients with distant lymph node metastasis by A) by chemotherapy response of metastatic, B) change of CEA level, C) complete macroscopic resection or not. (TIF 291 kb) [file 12885_2018_4998_MOESM4_ESM.tif]

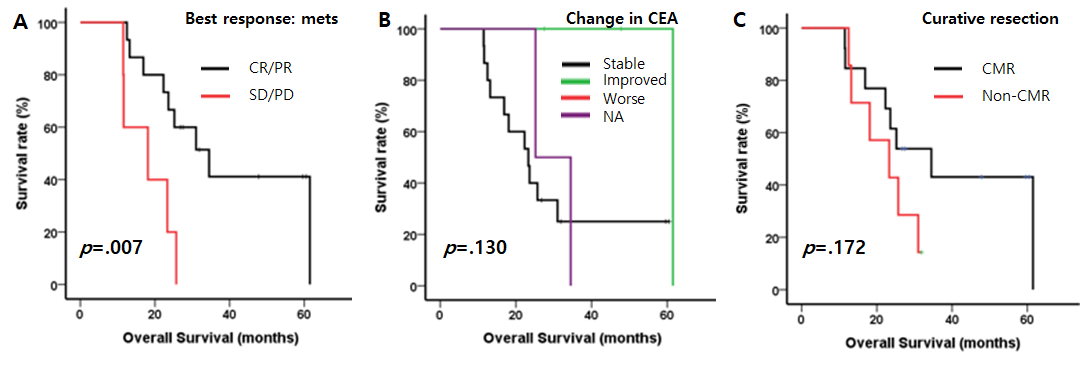

Supplement: Supplementary file 5 — Figure S5. Kaplan-Meier curves with log-rank test for overall survival of the patients with two or more distant metastasis by A) by chemotherapy response of metastatic site, B) change of CEA level, C) complete macroscopic resection or not. (TIF 452 kb) [file 12885_2018_4998_MOESM5_ESM.tif]
